# Deep learning-based pressure injury staging: a multicentre study involving 59 hospitals

**DOI:** 10.7189/jogh.16.04175

**Published:** 2026-06-19

**Authors:** Lu Zhou, Zhengyang Zhang, Junxia Wang, Hua Zhang, Changkun Zhong, Taohai Zong, Libing Sun, Man Luo, Lina Qiao, Hongyang Hu, Wei Zhang, Can Wang, Renhao Yang, Yujie Zhou, Ling Wang

**Affiliations:** 1 Department of Nursing, Peking University People’s Hospital, Beijing, China; 2 School of Nursing, Peking University, Beijing, China; 3 School of Nursing, Sun Yat-Sen University, Guangzhou, Guangdong, China; 4 The First Affiliated Hospital of Zhengzhou University, Zhengzhou, Henan, China; 5 The First Affiliated Hospital of Chongqing Medical University, Chongqing, China; 6 Department of Gastrointestinal Surgery, Peking University People's Hospital, Beijing, China; 7 Huailai County Hospital, Zhangjiakou, Hebei, China; 8 Trauma Centre, Peking University People’s Hospital, Beijing, China; 9 Wuhan Third Hospital, Wuhan, Hubei, China; 10 The First Affiliated Hospital of Xi’an Jiaotong University, Xi’an, Shaanxi, China; 11 Sir Run Run Shaw Hospital, Zhejiang University School of Medicine, Hangzhou, Zhejiang, China; 12 Hangzhou Yongliu Tech, Hangzhou, Zhejiang, China.; 13 Peking University Third Hospital, Beijing, China

## Abstract

**Background:**

Accurate assessment of pressure injury staging is essential for guiding appropriate care, reducing patient suffering, alleviating the healthcare burden, and improving quality of life. We aimed to develop a deep learning-based model for pressure injury recognition and to translate the best-performing model into a preliminary smartphone application.

**Methods:**

We conducted a multicentre retrospective study using pressure injury images collected from 59 hospitals. We applied to the data set three artificial intelligence-based image analysis models, Mask R-convolutional neural network (CNN) with ResNet-18, Mask R-CNN with Swin Transformer, and Segmenting Objects by Locations, version 2. We assessed model performance using standard evaluation metrics, including precision-recall curves, average recall (AR), average precision (AP), and mean average precision (mAP). The model achieving the best overall performance was selected for subsequent translation into a smartphone-based tool.

**Results:**

We included a total of 1903 pressure injury images, with 1713 used for model training and 190 for validation. Among the models evaluated, Mask R-CNN with Swin Transformer demonstrated the highest overall performance (mAP = 0.894, AP_50_ = 0.900, AP = 0.757, AR_100_ = 0.792), surpassing the corresponding metrics of the other two models. The model, based on Mask R-CNN with a Swin Transformer, was integrated into a smartphone for preliminary translation.

**Conclusions:**

The proposed deep learning-based system showed promising performance in pressure injury staging and may provide meaningful support for clinical decision-making. Future studies should focus on further validation and refinement using larger and more diverse data sets to enhance their applicability in routine clinical practice.

Pressure injury (PI) is defined as localised damage to the skin and/or underlying soft tissue, usually occurring over a bony prominence or in association with a medical device, resulting from intense or prolonged pressure, with or without shear forces [1]. A systematic review by Li *et al.* [2] reported a global prevalence of PI among hospitalised patients of 12.8%. The development of PI is associated with adverse health outcomes and substantial economic and healthcare burdens [3]. Specifically, PI increases the risk of patient mortality, prolongs hospital stays, and raises the likelihood of hospital readmission [4–6]. In the USA, the annual cost of PI has been estimated at USD 26.8 billion [7]. Management of PI largely depends on the lesion stage. Early-stage PI can often be addressed by relieving persistent local pressure, whereas advanced PI typically require additional interventions, such as specialised wound dressings [8]. In routine clinical practice, only a limited proportion of healthcare providers have received formal training in wound care [9]. Furthermore, patients with PI, particularly older adults with limited mobility, rarely seek hospital care specifically for this condition, and care is often provided by family members or other caregivers rather than healthcare professionals [10], potentially affecting PI recovery. Accurate assessment of PI stage is therefore important to support both medical staff and caregivers in identifying PI severity and guiding appropriate treatment decisions.

Deep learning may provide a supportive approach for healthcare professionals and caregivers in identifying PI stages. As a subfield of machine learning, deep learning enables complex information processing and has demonstrated strong performance in image-based analysis [11]. Previous studies have investigated deep learning methods for PI recognition and reported encouraging results. For instance, Liu *et al.* developed a deep learning model using PI images from four hospitals [12]. Seo *et al.* applied convolutional neural network (CNN) methods to PI staging [13], and Cho *et al.* used a vision transformer model to identify PI stages using 395 images from three hospitals [14]. Despite these efforts, several limitations remain. Some studies employed only a single model without comparison to alternative approaches, limiting the ability to identify the most effective method [15]. Others relied on images from only one or a limited number of hospitals, thereby restricting the diversity of data sources [14]. In addition, certain studies were based on relatively small sample sizes (≤500), which may affect model training and performance [16].

To address the limitations of previous studies and improve model performance in PI staging, we implemented and compared three deep learning models. We developed a comparative framework that covers algorithms ranging from lightweight convolutional neural networks to advanced Transformer architectures. We included Mask R-CNN with ResNet18 as a lightweight baseline to explore the feasibility of balancing efficiency and performance for instance segmentation in PI staging. We introduced Segmenting Objects by Locations, version 2 (SOLOv2), to address the irregular structure of PI wounds and to examine the applicability of the anchor-free paradigm to boundary reconstruction. We used Mask R-CNN with Swin Transformer to explore whether a global attention mechanism could improve feature extraction in regions with indistinct boundaries, such as peri-wound erythema. These three approaches are commonly used instance segmentation architectures in image recognition tasks. Mask R-CNN with ResNet18 is considered relatively stable and efficient to train [17]. Mask R-CNN with Swin Transformer has been reported to achieve relatively high training accuracy [14], and SOLOv2 offers relatively fast training speed with a simple model structure [12]. In addition, to increase both the quantity and quality of images used for model training, we collected more than 2000 PI images from 59 hospitals. These data were used to train the models to improve performance. Finally, the best-performing model was translated into a practical tool, providing a basis for future intelligent PI recognition.

Our primary aim was to train and evaluate three different deep learning approaches for PI recognition using images collected from hospitals, and to determine the best-performing model. Further, we aimed to preliminarily integrate the best-performing model into a smartphone application.

## METHODS

### Study design and data sources

We conducted an exploratory, retrospective observational analysis and reported it in accordance with the STROBE checklist. We collected PI images from 59 hospitals in China (Table S1 in the **Online Supplementary Document**). The images were obtained from patients during hospital visits after informed consent was obtained. To ensure standardised submission and efficient data management, we established an image upload platform prior to data collection, and all images were transmitted in encrypted form to ensure compliance with medical data regulations.

### PI staging judgment

According to the National Pressure Ulcer Advisory Panel staging system, PIs are classified as stage one (non-blanchable erythema), stage two (partial-thickness skin loss), stage three (full-thickness skin loss), stage four (full-thickness skin and tissue loss), unstageable (obscured full-thickness skin and tissue loss), and deep tissue injury [1].

### Requirements for PI images

To ensure suitability for deep learning training, we required that included PI images meet the following criteria: the image content depicted PI; resolution of at least 2106 × 1080 pixels; pixel density of at least 72 pixels per inch (PPI); colour depth of at least 8-bit red-green-blue; and image format in Joint Photographic Experts Group or Portable Network Graphics.

We did not have other uniform hardware requirements for image acquisition, but adequate lighting conditions were required during capture. We obtained images during patient visits to participating hospitals, with no restriction on anatomical site, provided that the wound was clinically identified as a PI. To prevent data leakage and potential bias during model training, we required that nursing experts at each hospital ensure that images of the same PI at the same site in the same patient were not uploaded multiple times. Before model training, if the PI images are not accurately labelled for any reason and this affects training, they were excluded.

### Process of PI image annotation and staging judgment

Annotation and staging of PI images were conducted in two stages. First, wound care specialists at each hospital annotated the images and determined the stage (specialists had at least 10 years of wound care experience and were capable of independent wound management). Subsequently, a specialised PI clinical team, composed of wound care experts, reviewed the annotations and staging provided by the hospital specialists to verify their accuracy. In cases of disagreement in annotation or staging, the images were further evaluated by a senior wound care expert in China to ensure annotation and staging accuracy.

### The algorithm process of the PI staging model

For PI image analysis we employed three deep learning models, Mask R-CNN with ResNet-18, Mask R-CNN with Swin Transformer, and SOLOv2. Mask R-CNN with ResNet-18 and Mask R-CNN with Swin Transformer are two-stage instance segmentation models, with backbone networks (a convolutional neural network and a transformer, respectively) responsible for feature extraction [18]. Mask R-CNN with ResNet-18 is computationally efficient and can achieve satisfactory performance with relatively limited training data [19], whereas Mask R-CNN with Swin Transformer generally achieves higher accuracy but requires greater computational resources and benefits from larger data sets [20]. SOLOv2 is a single-stage instance segmentation model that directly predicts instance masks based on object location and scale, offering faster inference compared with two-stage models such as Mask R-CNN [21]. Given these distinct characteristics, we comparatively evaluated these models to identify the optimal approach for PI detection and potential clinical application.

### The algorithm process of the PI model

Mask R-CNN (ResNet-18) and Mask R-CNN (Swin Transformer) share the same algorithmic workflow and differ only in their backbone networks for feature extraction, namely a convolutional ResNet-18 and a self-attention-based Swin Transformer, respectively. We implemented both models within the standard Mask R-CNN framework. The training process of Mask R-CNN consists of seven main steps: data input, feature extraction, multi-scale feature fusion, region proposal generation, region-of-interest alignment, classification and bounding-box regression, and mask prediction [22]. The algorithmic flow of SOLOv2 is relatively straightforward [23]. The input image is processed by a backbone network and a feature pyramid network to extract multi-scale features. A grid-based representation is then used to localise instances; Two parallel branches subsequently predict category scores and generate dynamic convolution kernels, which are applied to produce instance masks. We obtained final predictions after post-processing with Matrix NMS.

We randomly divided the data sets into training and validation sets at a 9:1 ratio using computer-based randomisation. We conducted model training using the stochastic gradient descent optimiser with an initial learning rate of 0.0001, momentum of 0.9, and weight decay of 0.0001 [22,24,25]. The gradient noise introduced by stochastic gradient descent during mini-batch optimisation may provide an implicit regularisation effect, guiding the model toward flatter minima in the loss landscape and thereby mitigating overfitting and improving generalisation in fine-tuning settings [26]. We determined the number of training epochs and iterations based on the sample size [24], and the batch size was calculated accordingly. We trained the model for 120 epochs (51390 iterations) with an effective batch size of four (two per graphics processing unit (GPU)) using data parallelism on two NVIDIA A100 (40G) GPUs (NVIDIA Corporation, Santa Clara, California, USA). We evaluated model training quality by monitoring loss curves [24], including classification loss (‘loss_cls’), bounding box regression loss (‘loss_bbox’), and mask loss (‘loss_mask’), which respectively reflect category prediction accuracy, localisation precision, and pixel-level segmentation performance.

### Model evaluation

We evaluated model performance using precision-recall (PR) curves, average recall (AR), average precision (AP), and mean average precision (mAP) [27]. Recall reflects the proportion of true positive instances correctly identified, whereas precision indicates the proportion of correct predictions among all positive predictions [27]. The primary performance metric was mAP, calculated as the mean AP across all categories, with higher values indicating better overall detection performance [15].

### Application of software in the research process

We conducted model training and validation using Python, version 3.8 (Python Software Foundation, Wilmington, Delaware, USA) and the PyTorch, version 1.12.1 (Meta Platforms, Inc., Menlo Park, California, USA) framework, with model development and training implemented using the MMDetection, version 3.3.0 (OpenMMLab, Shanghai, China) (Table S2 in the **Online Supplementary Document**).

We developed the front end of the intelligent PI assessment system using the native WeChat Mini Program framework (Tencent Holdings Ltd, Shenzhen, China), while the back end was built using the Java-based Spring Boot framework (VMware, Inc., Palo Alto, California, USA). We implemented the algorithm module in Python, and deployed the Mask R-CNN with the Swin Transformer model on an NVIDIA A100 GPU server to perform image segmentation and staging. The system used cloud-based object storage and a relational database to manage images and clinical data, enabling a closed-loop assessment process (Figure S1 in the **Online Supplementary Document**).

## RESULTS

We collected a total of 2637 PI images from 59 hospitals across China. Following quality screening, we excluded 672 images due to insufficient resolution or pixel density (resolution <2160 × 1080 or PPI<72). Prior to model training, we excluded an additional 62 images due to poor image quality, which resulted in nonstandard annotations, including mislabelling of peri-wound features, incomplete annotation of pigmentation or erythema with only the outer ring marked, overlap between wound bed boundaries and damaged areas, and excessively large annotated regions for wound boundaries. We included a total of 1903 images in the final analysis. We randomly divided the data set into training and validation sets at a 9:1 ratio, comprising 1713 and 190 images, respectively. PI staging by the deep learning models was primarily based on identifying tissue types in the images and on their distribution in the data set (**Table 1**; Figure S2 in the **Online Supplementary Document**).

**Table 1:** Pressure injury image tissue type distribution was included

Characteristics of skin tissue	Characteristics of the type	Labels	Images (training set), n	Images (validation set), n	Total, n
Suspected deep tissue injury area	Staging	area_2	207	21	228
Granulation tissue (black)	Staging	colour_1	986	107	1093
Slough (yellow)	Staging	colour_2	860	92	952
Eschar	Staging	colour_3	547	49	596
Exposed dermis	Staging	depth_1	284	41	325
Visible fat layer	Staging	depth_2	207	19	226
Fascia/muscle/tendon	Staging	depth_3	316	33	349

### Analysis of the model’s training process

**Figure 1:**
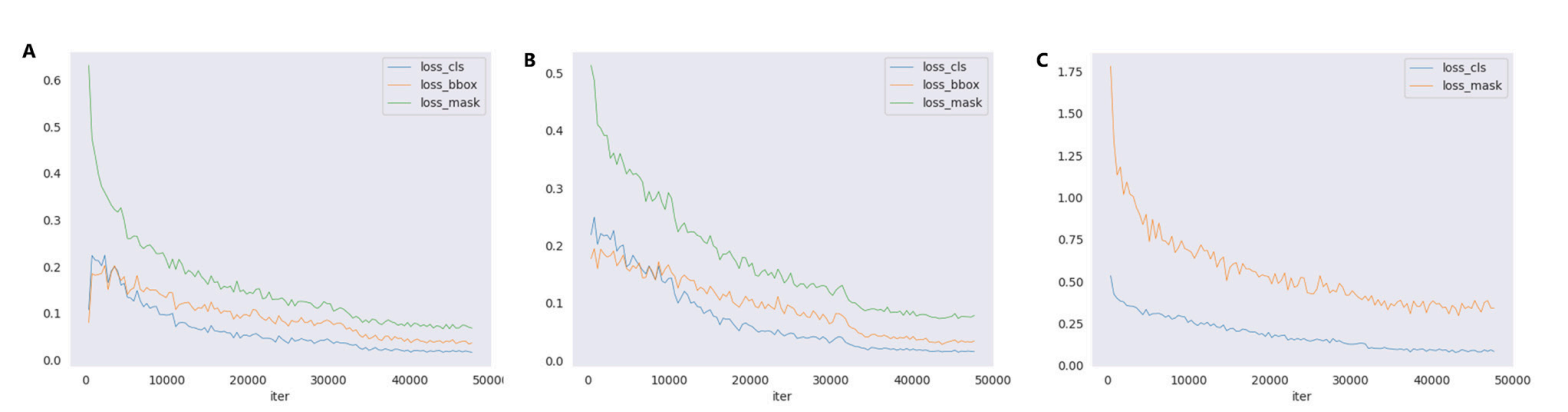
The variation of the loss curve during the model training process. **Panel A.** Mask R-CNN (Swin Transformer). **Panel B.** Mask R-CNN (ResNet-18). **Panel C.** SOLOv2. The x-axis represents the number of training iterations, and the y-axis represents the loss value.

The training dynamics of Mask R-CNN with ResNet-18 and Mask R-CNN with Swin Transformer were similar and showed stable convergence. For both models, the classification loss (‘loss_cls’) decreased rapidly during early training and then gradually plateaued. The bounding box regression loss (‘loss_bbox’) declined steadily with minimal fluctuation, and the mask loss (‘loss_mask’) decreased consistently throughout training. In contrast, SOLOv2 exhibited a slower decline in ‘loss_cls,’ and its ‘loss_bbox’ showed greater fluctuation during training (**Figure 1**, Panel A–C).

### Performance evaluation of PI staging models

**Figure 2:**
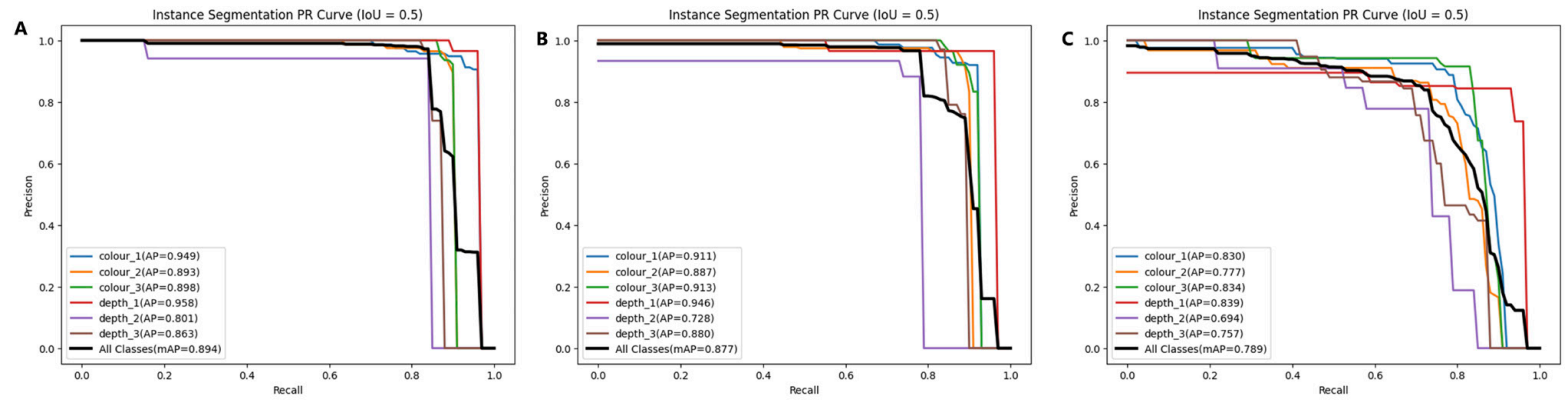
Relationship between model precision and recall. **Panel A.** Mask R-CNN (Swin Transformer). **Panel B.** Mask R-CNN (ResNet-18). **Panel C.** SOLOv2. AP – average precision, IoU – intersection over union, mAP – mean average precision, PR – precision-recall.

Mask R-CNN with ResNet-18 and Mask R-CNN with Swin Transformer achieved mA*P* values of 0.877 and 0.894, respectively, while SOLOv2 showed a lower mAP of 0.789 (**Figure 2**, Panel A–C).

We evaluated model performance using AP_50_, AP (0.5:0.95), and AR_100_. Mask R-CNN with Swin Transformer achieved the highest performance across all metrics (AP_50_ = 0.900, AP = 0.757, AR_100_ = 0.792). This was followed by Mask R-CNN with ResNet-18 (AP_50_ = 0.885, AP = 0.715, AR_100_ = 0.758). SOLOv2 showed comparatively lower performance (AP_50_ = 0.805, AP = 0.501, AR_100_ = 0.603). The PR curves shown were consistent with the quantitative results (**Table 2**; Figure S3 in the **Online Supplementary Document**).

**Table 2:** Evaluation indicators in the deep learning process of three models

Models	Epochs	Validation set, n	AP	AP_50_	AP_75_	AP_S_	AP_M_	AP_L_	AR_1_	AR_10_	AR_100_	AR_S_	AR_M_	AR_L_
Mask R-CNN (ResNet-18)	120	190	0.715	0.885	0.810	0.450	0.669	0.732	0.633	0.758	0.758	0.450	0.670	0.772
Mask R-CNN (Swin Transformer)	120	190	0.757	0.900	0.838	0.496	0.633	0.668	0.782	0.792	0.792	0.500	0.682	0.808
SOLOv2	120	190	0.501	0.805	0.531	0.100	0.348	0.632	0.485	0.598	0.603	0.394	0.347	0.633

IoU – intersection over union, AP – average precision, AP_50_ – average precision at intersection over union = 0.50, AP_75_ – average precision at intersection over union = 0.75, AP_S_ – average precision for small objects, AP_M_ – average precision for medium objects, AP_L_ – average precision for large objects, AR_1_ – average recall, max one detection per image, AR_10_ – average recall, max 10 detections per image, AR_100_ – average recall, max 100 detections per image, AR_S_ – average recall for small objects, AR_M_ – average recall for medium objects, AR_L_ – average recall for large objects, Mask R-CNN – Mask R-convolutional neural network, SOLOv2 – Segmenting Objects by Locations version 2

### Clinical transformation of Mask R-CNN (Swin Transformer) models

We implemented the best-performing Mask R-CNN with Swin Transformer model in a PI staging recognition smartphone application. The application features two operational modes – a medical staff interface and a patient interface. Through the medical staff interface, clinicians can capture PI images and receive artificial intelligence-assisted stage predictions. The patient interface allows patients to upload PI images for automated staging. The platform also supports remote interaction between patients and clinical staff, enabling image-based consultations and follow-up monitoring. Selected screenshots of the application interface were provided (Figure S4 in the **Online Supplementary Document**).

## DISCUSSION

**Figure 3:**
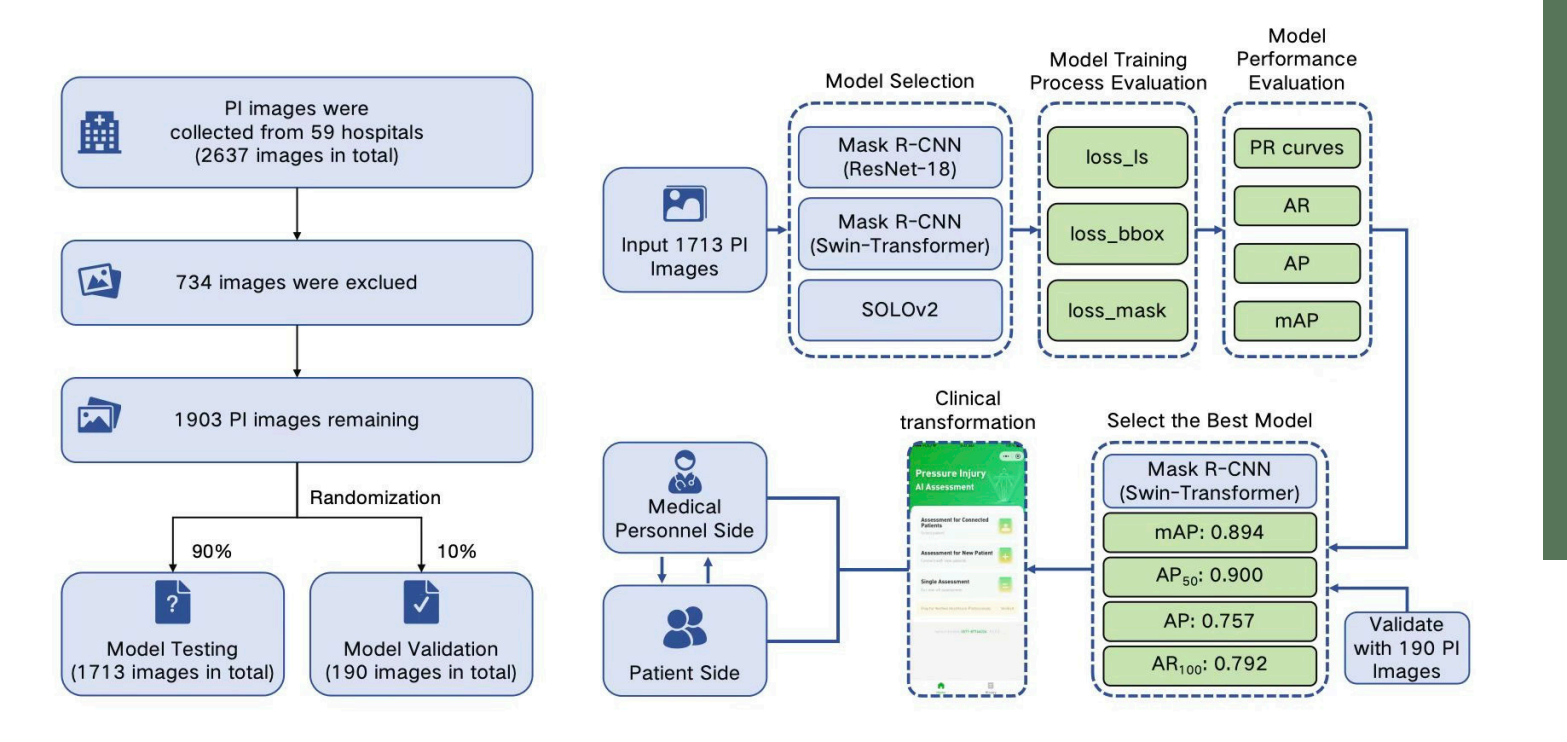
Summary graphic illustration of data collection, model training, model evaluation and clinical application. AP – average precision, AR – average recall, CNN – convolutional neural network, loss_bbox – bounding box regression loss, loss_cls – classification loss, loss_mask – mask loss, mAP – mean average precision, PI – pressure injury, PR – precision-recall, SOLOv2 – segmenting objects by locations, version 2.

We established a retrospective data set comprising over 2000 PI images from 59 hospitals and developed three deep learning-based PI staging models, which we systematically compared on this data set. The model with the best performance was subsequently implemented in a PI staging recognition application, demonstrating the feasibility of translating such models into clinical practice. This deep learning-based PI staging tool may serve as an auxiliary reference for PI stage assessment and support more standardised PI management. In settings with limited access to specialised wound care professionals, the tool could assist clinical staff and caregivers in identifying PI stages and inform routine decision-making [28]. By enabling stage identification, the tool may also help patients and caregivers better understand PI severity and adopt stage-appropriate management strategies, thereby potentially preventing further deterioration and reducing associated healthcare costs [7]. Overall, the PI staging recognition tool developed in this study may be of practical relevance to clinical staff, patients, and the healthcare system, particularly in resource-limited settings. A summary illustration of data collection, model training, model evaluation, and clinical application was provided (**Figure 3**).

We compared performance of three instance segmentation models for PI staging using training loss curves and multiple evaluation metrics, including AP, AR, mAP, and PR curves. Overall, Mask R-CNN (Swin Transformer) showed higher performance across the evaluated metrics compared with Mask R-CNN (ResNet-18) and SOLOv2. The improved performance of Mask R-CNN (Swin Transformer) may be related to the characteristics of its transformer-based backbone, which has been reported to capture broader contextual information in image analysis tasks. Such properties may be beneficial for PI images, where lesion boundaries, tissue heterogeneity, and surrounding skin features are often complex. Similar findings have been reported by Brehmer *et al.* [29], who observed effective PI stage identification using transformer-based deep learning approaches. Mask R-CNN (ResNet-18) demonstrated slightly lower performance, which may be associated with the inherent limitations of convolutional neural networks in modelling long-range contextual information in complex visual scenes [24]. In contrast, SOLOv2 showed comparatively lower performance across multiple metrics. This may be related to its single-stage instance segmentation framework, which has been reported to be less effective for tasks involving small, irregular, or densely distributed targets, such as PI lesions [23]. Consistent results have also been observed in other medical imaging applications, including the study by Park *et al.* [30], where Mask R-CNN outperformed SOLOv2. Taken together, the findings of this study suggest that Mask R-CNN with a Swin Transformer backbone may be a suitable option for PI staging tasks. This approach may support more standardised and reproducible PI stage assessment and provide a methodological basis for further exploration of deep learning-assisted PI management in clinical and community healthcare settings.

### Implications for clinical and future practice

Based on data collected from multiple hospitals, this study developed an optimal PI staging recognition tool using several deep learning approaches. This tool has not yet been implemented in clinical practice, but it may help address the shortage of wound care specialists in some regions, support non-specialist staff in more accurately identifying PI stages, promote earlier healing, and reduce patient suffering. In addition, the tool may be used by patients and their caregivers, enabling PI staging at home and facilitating appropriate wound management, thereby helping reduce the healthcare burden. However, several barriers to implementation should be considered. First, the model was developed using images from individuals with yellow skin tones, and its applicability to other skin tones remains uncertain. Second, further training on larger data sets is needed to improve model performance, ideally to a level at least as good as that assessed by wound care specialists. Finally, reliance on smartphone-based assessment rather than evaluation by wound care specialists may affect user trust in the tool.

### Guidance on future research directions

The application has not yet been implemented in clinical practice. Future work will continue to collect PI images to further train the model and improve staging accuracy. In addition, multicentre external validation will be conducted to assess generalizability. Randomised controlled trials are also planned to evaluate whether the application is non-inferior to assessment by wound care specialists in PI staging. Finally, wound care experts will be engaged to systematically summarise care pathways for different PI stages and integrate them into the application, enabling stage-based, personalised care recommendations and forming a closed-loop decision-support system.

### Research limitations

Although we included nearly 2000 samples, which is higher than the sample size in most current studies on PI recognition, there is currently no established evidence regarding the required sample size for PI recognition tasks in deep learning. Due to technical and resource constraints, we reported model performance using point estimates only, without 95% confidence intervals, which may limit the precision of interpretation. All included samples were from individuals with yellow skin tones, caution should be exercised when applying this tool to other populations, and its applicability requires further validation. The training process for deep learning models is inherently opaque, and the possibility of overfitting cannot be entirely ruled out. Given that the accuracy of PI staging is closely related to the size of the training data set, we used 90% of the data for training, 10% for validation, and performed no external validation, which may affect the assessment of model generalisability.

## CONCLUSIONS

Mask R-CNN (Swin Transformer) demonstrated favourable performance in PI staging and was translated into a smartphone-based application. Future research should focus on expanding the training data set to further improve model performance and exploring the integration of stage-specific, patient-centred care recommendations based on PI staging and individual clinical characteristics.

## Additional material


Online Supplementary Document
