# Supplementary material for: Deep learning-based pressure injury staging: a multicentre study involving 59 hospitals
Source: J Glob Health. 2026 Jun 19;16:04175. doi: 10.7189/jogh.16.04175 (PMC13281261; doi:10.7189/jogh.16.04175)
Supplement: Online Supplementary Document [file jogh-16-04175-s001.pdf]

Supplement to: Zhou L, Zhang Z, Wang J, Zhang H, Zhong C, Zong T, Sun L, Luo M, Qiao L, Hu H, Zhang W, Wang C, Yang R, Zhou Y. Deep learning-based pressure injury staging: a multicentre study involving 59 hospitals. J Glob Health. 2026;16:04175.

Table S1: The source of images of pressure injuries

Number	Hospital Name (English)	Number of pressure injuries images
1	The First Affiliated Hospital Of Zhengzhou University	226
2	Baoji Central Hospital	130
3	HUNAN CANCER HOSPITAL	128
4	Peking University Third Hospital	99
5	THE SECOND HOSPITAL OF DALIAN MEDICAL UNIVERSITY	76
6	General Hospital of Ningxia Medical University	76
7	Shandong Provincial Hospital	73
8	Haikou People's Hospital	71
9	HEBEI GENERAL HOSPITAL	66
10	Henan Provincial People's Hospital	64
11	AFFILIATED HOSPITAL OF JINING MEDICAL UNIVERSITY	63
12	The First Affiliated Hospital of Chongqing Medical University	62
13	Xinjiang Uygur Autonomous Region Hospital of Traditional Chinese Medicine	61
14	THE FIRST AFFILIATED HOSPITALZHEJIANG UNIVERSITY SCHOOL OF MEDICINE	59
15	Affiliated Hospital of Zunyi Medical University	58
16	Chongqing Emergency Medical Center	58
17	THE AFFILIATED HOSPITAL OF QINGDAO UNNVERSITY	57
18	PEKING UNIVERSITY PEOPLE'S	56

	HOSPITAL	
19	Liwan District People's Hospital of Guangzhou	51
20	WUHAN THIRD HOSPITAL	51
21	THE FEOPLES HOSPITAL OF GUANGXI ZHUANG AUTONOMOUS REGION	50
22	Peking University International Hospital	49
23	Puyang Oilfield General Hospital	49
24	Shenzhen Luohu Hospital Group Luohu People's Hospital	48
25	SIR RUN RUN SHAW HOSPITAI ZHEIANG UNIVERSITY SCHOOL OF MEDICINE	46
26	The First Affiliated Hospital of Xi'an Jiaotong University	46
27	The Affiliated Hospital of Shandong University of Traditional Chinese Medicine	42
28	THE AFFILIATED HOSPITAL OF SOUTHWEST MEDICAL UNIVERSITY	41
29	The Affiliated Central Hospital of Shandong First Medical University	40
30	SHENZHEN SECOND PEOPLE'S HOSPITAL	36
31	CHONGQING UNIVERSITY THREE GORGES HOSPITAL	33
32	THE PEOPLE'S HOSPITAL OF LESHAN	32
33	SHANGHATPUDONG HOSPITAI	31
34	Xiangya Hospital of Central South University	31
35	General Hospital of Tianjin Medical University	30

36	First Hospital of Qiqihar City	30
37	ZHE JIAMG CANCER HOSPITAL	27
38	The 900 Hospital of the Joint Service Support Force of the People's Liberation Army of China	27
39	Fujian Provincial Hospital	26
40	The 903rd Hospital of the Joint Logistics Support Force of the Chinese People's Liberation Army	25
41	Beijing Yanhua Hospital	24
42	The First Affiliated Hospital of Harbin Medical University	23
43	The Affiliated Hospital of Binzhou Medical University	23
44	ZHEIIANG PROVINCIAL PEOPLE'S HOSPITAI	22
45	XIANGYA CHANGDE HOSPITAL	22
46	The 988th Hospital of the Joint Logistics Support Force of the Chinese People's Liberation Army	22
47	Weifang Hospital of Traditional chinese Medicine	21
48	Strategic Support Force Specialty Medical Center	20
49	The Second Affiliated Hospital of Nanjing Medical University	19
50	Shanxi Provincial Children's Hospital	18
51	BeiJing SHIJITAN HOSPITAL	16
52	CHINA JAPAN FRIENDSHIP HOSPTAL	15
53	THE FIRST HOSPITAL OF CHINA MEDICAL UNIVERSITY	13
54	Dongzhimen HospitalBeijing University of	13

	Chinese Medicine	
55	PEKING UNIVERSITY FIRST HOSPITAL	11
56	First Affiliated Hospital of Xinjiang Medical University	11
57	Zhongshan Hospital	9
58	The Seventh Medical Center of the General Hospital of the Chinese People's Liberation Army	8
59	SIR RUN RUN SHAW HOSPITAI ZHEIANG UNIVERSITY SCHOOL OF MEDICINE	3

Table S2: Software name and version number during model training and validation

**Software**	**Version**
Ubuntu	18.04
NVIDIA GPU Driver	455.0
CUDA	11.0
CUDNN	8.0.4
Python	3.8
Pytorch	1.12.1
Anaconda3	2024.06-1
MMDetection	3.3.0
MMCV	2.1.0
MMEngine	0.8.4
numpy	1.24.3
matplotlib	3.7.5
scipy	1.10.1
shapely	2.0.3
pycocotools	2.0.7
terminaltables	3.1.10
tqdm	4.66.1
opencv-python	4.8.1.78
Pillow	10.1.0
scikit-learn	1.3.2
albumentations	1.3.1

Figure S1: Development process of the pressure injuries recognition mobile application

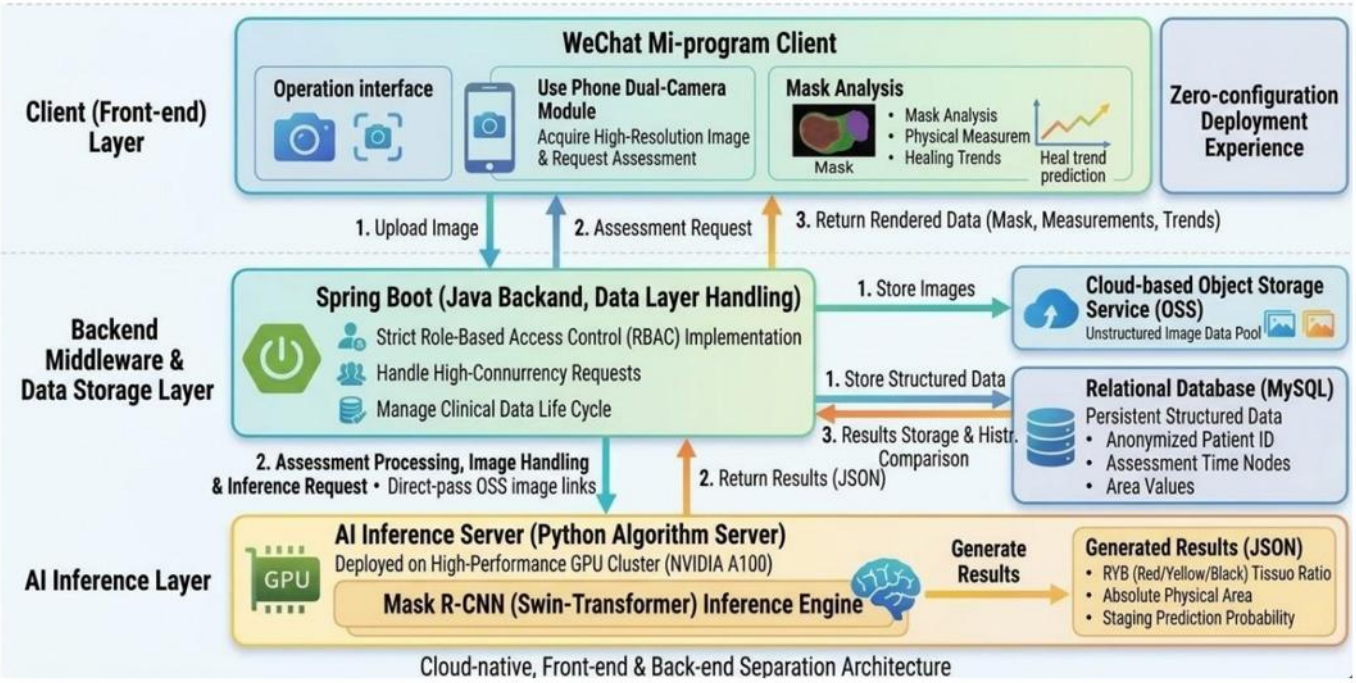

Figure S2: The logic of judging pressure injuries staging in the process of deep learning

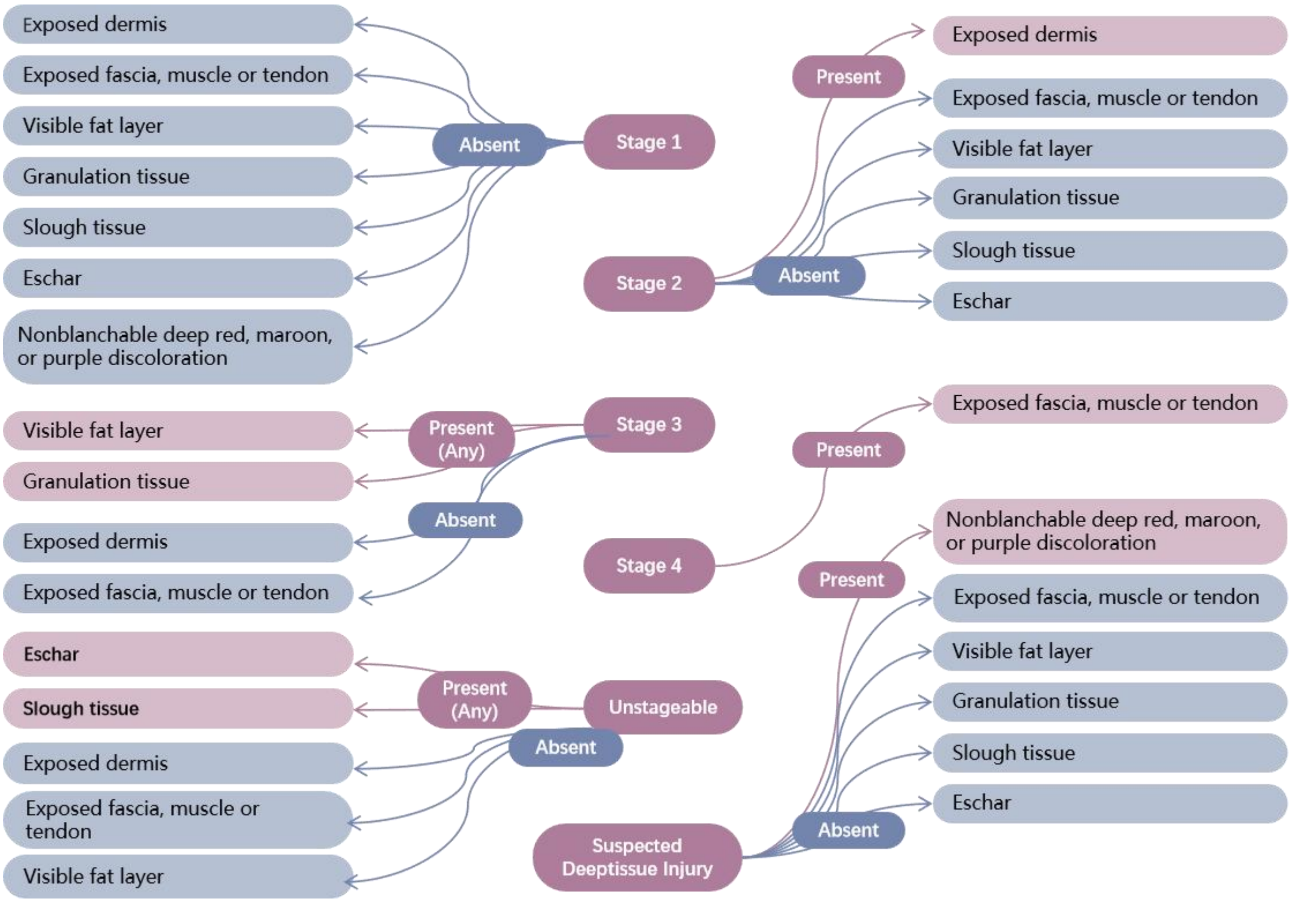

Figure S3: Precision Recall curves of the three models (Mask-rcnn_swin-t-p4-w7: Mask R-CNN (Swin-Transformer); Solov2-light_r18: SOLOv2; Mask-rcnn_r18: Mask R-CNN (ResNet-18)) PR- precision-recall

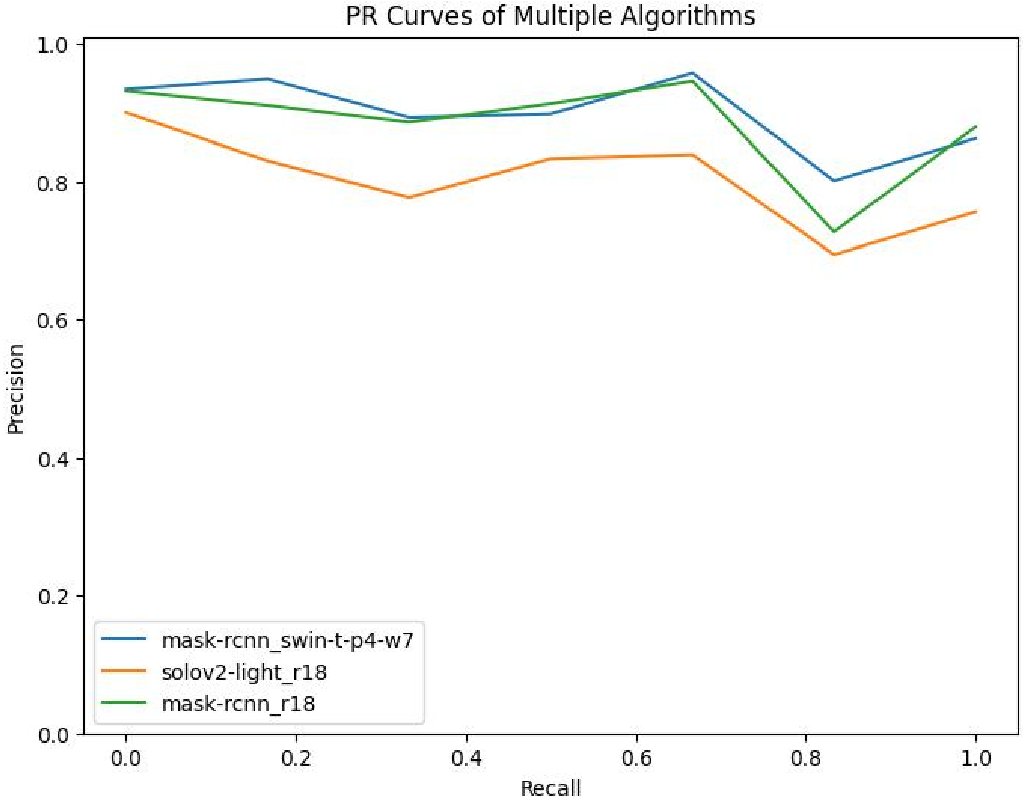

Figure S4: Part of the interface of the pressure injuries stage recognition tool

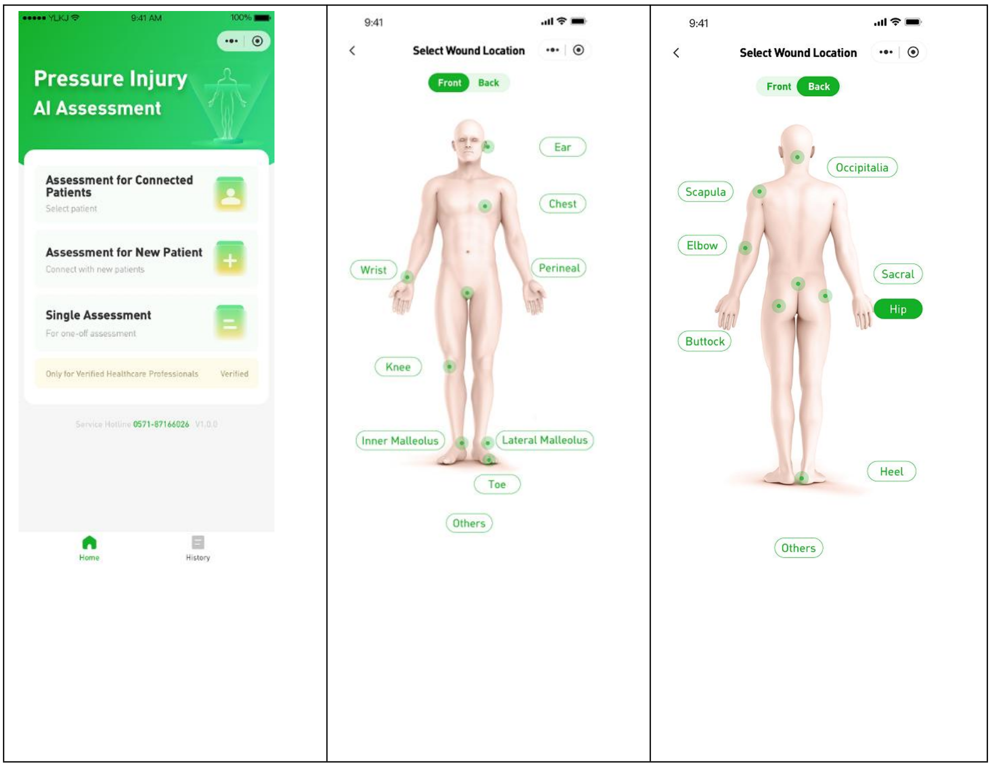

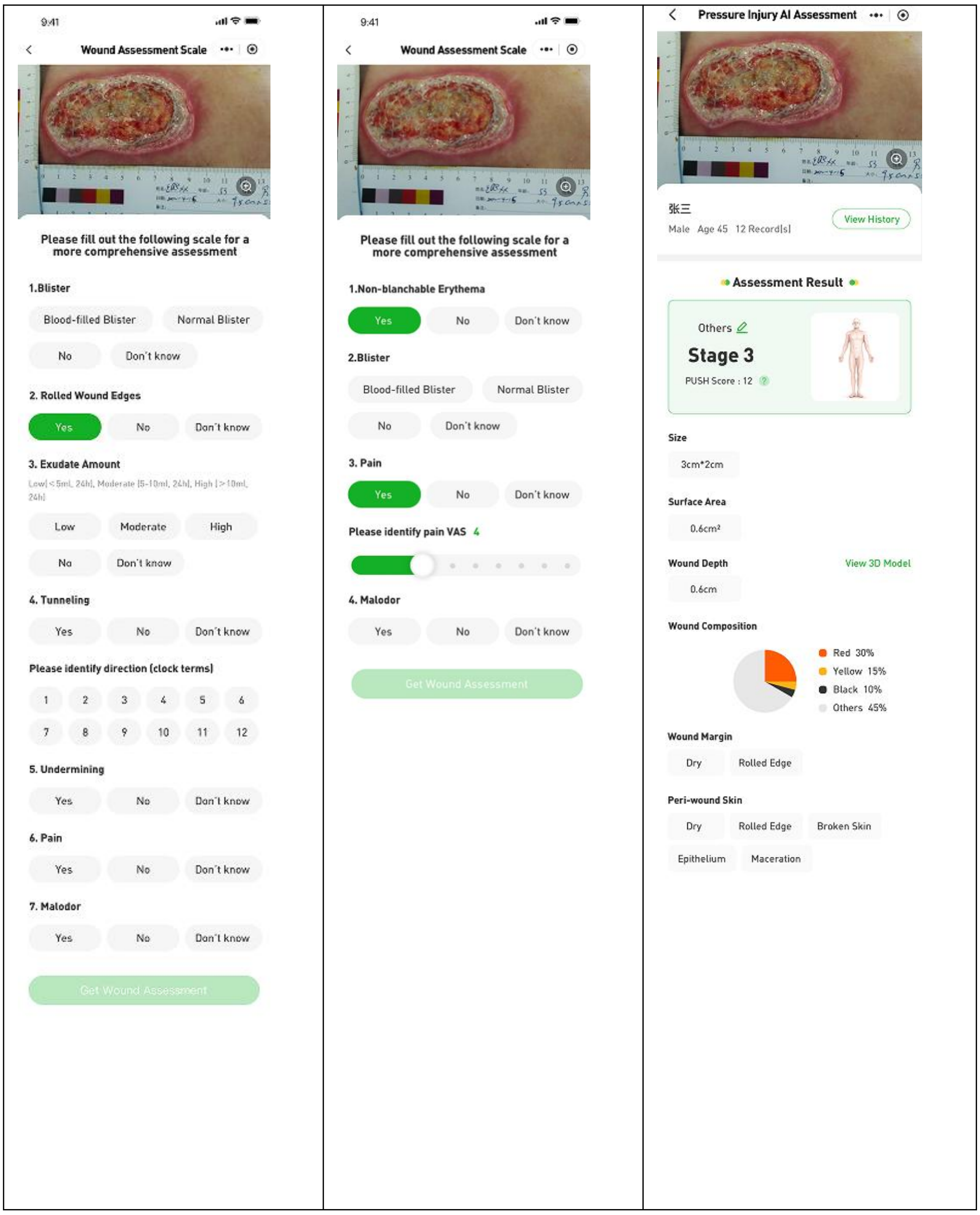
9:41
Wound Assessment Scale
Please fill out the following scale for a more comprehensive assessment
1.Blister
Blood-filled Blister
Normal Blister
No
Don't know
2. Rolled Wound Edges
Yes
No
Don't know
3. Exudate Amount
Low(<5ml, 24h), Moderate (5-10ml, 24h), High (>10ml, 24h)
Low
Moderate
High
No
Don't know
4. Tunneling
Yes
No
Don't know
Please identify direction (clock terms)
1 2 3 4 5 6
7 8 9 10 11 12
5. Undermining
Yes
No
Don't know
6. Pain
Yes
No
Don't know
7. Malodor
Yes
No
Don't know
Get Wound Assessment
9:41
Wound Assessment Scale
Please fill out the following scale for a more comprehensive assessment
1.Non-blanchable Erythema
Yes
No
Don't know
2.Blister
Blood-filled Blister
Normal Blister
No
Don't know
3. Pain
Yes
No
Don't know
Please identify pain VAS 4
4. Malodor
Yes
No
Don't know
Get Wound Assessment
Pressure Injury AI Assessment
张三
Male Age 45 12 Record(s)
View History
Assessment Result
Others
Stage 3
PUSH Score : 12
Size
3cm*2cm
Surface Area
0.6cm²
Wound Depth
View 3D Model
0.6cm
Wound Composition
Red 30%
Yellow 15%
Black 10%
Others 45%
Wound Margin
Dry
Rolled Edge
Peri-wound Skin
Dry
Rolled Edge
Broken Skin
Epithelium
Maceration
